# Rab3D Is Critical for Secretory Granule Maturation in PC12 Cells

**DOI:** 10.1371/journal.pone.0057321

**Published:** 2013-03-19

**Authors:** Tanja Kögel, Rüdiger Rudolf, Erlend Hodneland, John Copier, Romano Regazzi, Sharon A. Tooze, Hans-Hermann Gerdes

**Affiliations:** 1 Department of Biomedicine, University of Bergen, Bergen, Norway; 2 Interdisciplinary Center of Neurobiology, University of Heidelberg, Heidelberg, Germany; 3 London Research Institute Cancer Research United Kingdom, Lincoln's Inn Fields Laboratories, London, United Kingdom; 4 Department of Fundamental Neurosciences, University of Lausanne, Lausanne, Switzerland; University of Iowa, United States of America

## Abstract

Neuropeptide- and hormone-containing secretory granules (SGs) are synthesized at the *trans*-Golgi network (TGN) as immature secretory granules (ISGs) and complete their maturation in the F-actin-rich cell cortex. This maturation process is characterized by acidification-dependent processing of cargo proteins, condensation of the SG matrix and removal of membrane and proteins not destined to mature secretory granules (MSGs). Here we addressed a potential role of Rab3 isoforms in these maturation steps by expressing their nucleotide-binding deficient mutants in PC12 cells. Our data show that the presence of Rab3D(N135I) decreases the restriction of maturing SGs to the F-actin-rich cell cortex, blocks the removal of the endoprotease furin from SGs and impedes the processing of the luminal SG protein secretogranin II. This strongly suggests that Rab3D is implicated in the subcellular localization and maturation of ISGs.

## Introduction

SGs of neuroendocrine cells store neuropeptides and hormones until an adequate stimulus triggers their regulated exocytosis. In PC12 cells, newly formed ISGs move from the TGN [1] in a fast and microtubule-dependent manner to the cellular cortex, where they distribute in an F-actin and myosin Va dependent manner [2], [3], and complete maturation within a few hours [2], [4]. The maturation process of ISGs comprises homotypic fusion [5], luminal acidification and condensation [4], [6], processing of prohormones and neuropeptides [7], [8], and removal of membrane and proteins via clathrin-coated ISG-derived vesicles (IDVs) [9], [10], [11], [12], [13]. To date, the underlying mechanisms regulating these processes are poorly understood.

In search for proteins involved in these processes, we previously demonstrated that myosin Va, which restricts SGs to the peripheral F-actin cortex [3], is essential for SG maturation [14]. As myosin Va does not bind directly to membranes [15], linker proteins are necessary to connect myosin Va to neuroendocrine SGs. Such proteins were first described for melanosomes, the secretory organelles of melanocytes: myosin Va binds *via* melanophilin (also called synaptotagmin-like protein lacking C2 domains (Slac) 2-a) to Rab27A, which in turn is anchored to the melanosome membrane. This complex is necessary for the capture and distribution of melanosomes in the F-actin cortex [16], [17]. Similar composites were found for retinal pigment epithelial and pancreatic beta-cells, where MyRIP (Slac 2-c) and rabphilin-3A/granuphilin a/b were bound to Rab27A, respectively [18], [19]. It is therefore conceivable that transient complexes of similar composition could be involved in myosin Va-dependent ISG maturation [20], [21]. These complexes may not only differ with respect to the synaptotagmin-like component but may also encompass another rab protein.

In an attempt to identify the relevant Rab proteins for SG transport to the F-actin rich cortex, a systematic screen was performed on isoforms of Rab1 to 41 by expressing them as GFP fusion proteins in PC12 cells [22]. This revealed that only Rab3 and Rab27 were predominantly targeted to and essential for SG localization [22]. These data are in agreement with further studies showing that Rab3 and Rab27 isoforms are specifically targeted to SGs of PC12 cells [22], [23], [24]. Therefore, Rab3 and Rab27 isoforms are the most likely candidates for a role in ISG maturation. Since Rab27 has been suggested as a sensor for late maturation stages of secretory organelles [25], [26], we have investigated a possible role of Rab3 isoforms and provide evidence that Rab3D mediates a distinct maturation step of SGs.

## Materials and Methods

### Chemicals, antibodies, cDNAs

Reagents were purchased from Amersham (Piscataway NJ, USA), BD (Le Pont de Claix, France), BioRad (Hercules, CA, US), Fluka (Buchs, Germany), Invitrogen (Carlsbad, CA, US), J.T. Baker (Deventer, Holland), Merck (Darmstadt, Germany), Neuform (Lüneburg, Germany), Roth (Karlsruhe, Germany), Serva (Heidelberg, Germany), and Sigma (Steinheim, Germany and Saint-Louis, MO, US). Constructs pcDNA3-hCgB-GFP(S65T) [3] and pcDNA3-hCgB-EGFP [2] were described previously. The generation of the pcDNA3 plasmids encoding myc-Rab3A, B, C and D and the corresponding (N135I) mutants has been described previously [27]. Construct pRC/CMV PC2 (originally from Prof. N. Sediah) and the antibody against p18, the cleavage product of SgII were described previously [5]. Constructs pCMV2-FLAG and pCMV2-FLAG-MCLT (referred to as FLAG-myoVa-tail) and polyclonal antibodies Dil2 [28], [29] were kindly provided by Dr. J. A. Hammer III (NIH, Bethesda, USA). Bovine furin (bfurin) cDNA was kindly provided by Dr. W. Garten (Dept. of Virology, Univ. of Marburg, Germany). Monoclonal antibody mon148 against bfurin was kindly provided by Dr. J. Creemers (K. University of Leuven, Belgium). Polyclonal antiserum D2 was raised against GFP-peptide D2 [30]. Monoclonal antibody M5 against FLAG-epitope was purchased from Sigma. Secondary antibodies goat anti-rabbit TRITC, goat anti-mouse TRITC, goat anti-mouse FITC, goat anti-mouse Cy5, goat anti-rabbit rhodamine and goat anti-rabbit HRP were purchased from Jackson Immuno Research Labs (West Grove PA, USA).

### Cell culture and transfection

PC12 cells (rat pheochromocytoma 12 cells, clone 251) [31] were grown in DMEM, 10% horse serum (Gibco/Invitrogen, Karlsruhe, Germany) and 5% fetal calf serum (PAA, Pasching, Austria) at 37 °C/10% CO_2_. Cells were transfected by electroporation as previously described [30]. Expression of the transgenes under the control of cytomegalo virus (CMV) promotor was increased when indicated by incubation in medium supplemented with 10 mM sodium butyrate for 17.5 hours. PC12 cells were plated on poly-L-lysine-coated (PLL, 0.1 mg/ml) cell culture dishes or coverslips and fixed in 4% paraformaldehyde (PFA)/4% sucrose/PBS if not indicated differently.

### Pulse/chase-like protocols

Two different pulse/chase-like protocols were used as published before [14]. A short protocol was applied to analyze the biosynthetic transport of bfurin along the secretory pathway prior to its steady state distribution. To monitor the removal of bfurin from maturing SGs, cells were cotransfected with bfurin and hCgB-EGFP as a marker for SGs followed by incubation at 37°C for 2 h. Subsequently, cells were incubated at 20°C for 2 h (pulse), which blocked ISG formation and, as a consequence, led to the accumulation of bfurin and fluorescent hCgB-EGFP in the TGN. To release the temperature block, the cells were incubated at 37°C for different periods in culture medium as indicated (chase). It is of note that during the last 30 min of the block and during the chase the medium was supplemented with 10 µg/ml cycloheximide to preclude the arrival of newly synthesized hCgB-EGFP at the TGN [2]. This protocol allowed to monitor selectively the removal of bfurin from ISGs, before the main fraction of bfurin was distributed to the endosomal pathway via the plasma membrane, which would have made a discrimination of ISGs from endosomes very difficult. The disadvantage of this protocol for other purposes is that, due to the short expression time only weak fluorescence signals of the SG-marker and cotransfected proteins were.

A long pulse/chase-like protocol was used to produce fluorescent ISGs with high signal intensity. For this purpose cells were transfected with hCgB-GFP(S65T) [30] followed by incubation at 37°C for 5 to 24 h and subsequently for 17.5 h in the presence of sodium butyrate to enhance the expression of the GFP fusion protein. The biogenesis of SGs was subsequently blocked by incubation at 20°C for 2 h (pulse). Notably, only at this low temperature hCgB-GFP(S65T) is converted to its fluorescent form and accumulates in the TGN. Upon release of the temperature block in culture medium at 37°C for different time periods (chase) brightly fluorescent ISGs are formed, while newly translated hCgB-GFP(S65T) remains non-fluorescent.

### Fluorescence labeling

Indirect immunofluorescence labeling of cells was performed as described previously [30]. bfurin, FLAG and myc were stained with anti-furin (mon 148), anti-FLAG (M5), and anti-myc (9E10) antibodies, respectively, and subsequently with secondary antibodies coupled to fluorescent dyes. F-actin was fluorescently labeled with a phalloidin-TRITC conjugate (250 nM). Nuclei were stained with Hoechst®-dye.

### Determination of expression levels of myc-Rab3 fusion proteins

Transfected cells were immunostained against the myc-tag and imaged with a Zeiss Axiovert 200 microscope equipped with a 40× EC Plan Neofluoar NM 1. 3 objective, Polychrome V monochromator (TILL Photonics), CCD camera sensicam imago-QE (PCO) and TillVision 4. 0 software (Till Photonics). It is of note that random frames were chosen in the Hoechst®-dye channel. The data were acquired as 16-bit TIF-images. Immunofluorescence signals were quantitated by an in-house implemented MatLab® application (www.mathworks.com). Boundaries of cells were outlined by using the drawing tool of this application and used to measure and calculate the mean fluorescence intensity inside the boundaries. Background fluorescence levels were obtained by averaging the fluorescence intensity of 10 non-expressing cells per frame. The fluorescence intensity above background of all cells with normal S-phase nuclei was quantitated.

### Analysis of colocalization

To quantitate the colocalization of SGs with the F-actin rich cell cortex, images of cells were taken with a Leica TCS 4D confocal microscope (format 512×512 pixels). Colocalizing SGs were counted in 3D using IPLab 3.2.2 software for at least six cells/condition. To quantitate the colocalization of SGs with bfurin, optical sections throughout the cells (format at least 256×256 pixels) were taken at distance <250 nm with a Leica SP2 or SP5 confocal microscope equipped with a 63 x/1.4 NA PL APO oil objective. After binarization of the images the TGN was visible as a continuous extensive fluorescence signal of hCgB-GFP and furin in the perinuclear region and SGs as peripheral punctuate fluorescence signals with 3–20 pixels in diameter in (xy)-plane and ≥2 pixels in z-plane. Punctuate signals that did not meet these size criteria or were in continuity with the TGN signal were excluded from the evaluation. All counted SGs that displayed an overlap between the hCgB-GFP and bfurin signals of ≥3 pixels in (x-y)-dimension and ≥2 pixels in z-dimension were classified as co-localizing as described before [2], [14]. For evaluation, the hCgB-GFP signals were categorized as “colocalizing”, “non-colocalizing”, “below critical size” or “part of TGN”. To analyze the colocalization of SGs with Rab3D or Rab3D(N135I), preparations of SGs were spun down at 100 000 g onto coverslips placed into plasticine-leveled centrifuge tubes using a SW-40 rotor in a Beckman L-70 ultracentrifuge. Samples were fixed and stained against the myc-tag. Confocal 3D images (format of 512×512 pixels) were acquired at a z-plane distance of 500 nm using a Leica SP5 confocal microscope equipped with a 100 x/1.4 NA PL APO oil objective. Maximum projections, rendered with Leica software, were used to automatically quantitate the percentage of colocalization of punctuate signals of hCgB-GFP(S65T) and punctuate signals of anti-myc staining (MatLab® application). The lower grey scale cut and contrast enhancement were adjusted manually for each experiment and then applied to all conditions.

### Analysis of SGs by sucrose density gradient equilibrium cenrifugation

Cells were cotransfected with hCgB-EGFP and FLAG, FLAG-MyoVa-tail, myc-Rab3D or myc-Rab3D(N135I) and then cultured for two days including 17.5 h of sodium butyrate induction. Thereafter cells were resuspended in HBS buffer (10 mM Hepes/KOH to pH 7. 2/0. 25 M sucrose, 1 mM Mg(Ac)_2_, 1 mM EDTA, protease inhibitors: aprotinin 1 µg/ml, leupeptin 5 µg/ml, PMSF 0.5 mM, pepstatin 1 µg/ml, antipain 1 µg/ml, α_2_-macroglobulin 10 mU/ml, jodacetamide 18 µg/ml, benzamidine 1 mM) [32] and a postnuclear supernatant (PNS) was prepared by mechanical cracking of the cells and removal of nuclei by centrifugation. The PNS was then centrifuged for 10 min at 14 000 g (Beckman rotor 120.1). The resulting supernatant was centrifuged for 20 min at 100 000 g (Beckman rotor 120.1) to sediment SGs. The pellet was then resuspended in 100 µl HBS and subjected to equilibrium sucrose density gradient centrifugation into a step gradient with 0. 1 M steps at 25 000 rpm for 16 h (approx. 50 000–110 000 g, Beckman rotor SW-40). Aliquots of the gradient fractions were subjected to SDS-PAGE followed by Western blotting [30].

### Analysis of SgII-processing

Cells were transfected with pRC/CM-PC2, pcDNA3-myc-Rab3D, pcDNA3-myc-Rab3D(N135I) or pCMV2-FLAG. After sodium butyrate induction, the cells, grown in 60 mm dishes, were incubated for 30 min with SO_4_-free medium and then pulse-labeled for 1 h with 2 ml of medium containing 3 mCi [^35^S]sulphate. Then cells were washed twice with 1 ml medium containing 1. 6 mM Na_2_SO_4_, followed by a 3 h chase in 3 ml medium containing 1.6 mM Na_2_SO_4_. Thereafter, cells were washed twice with PBS pH 7.4 and then incubated for 20 min at 4°C in lysis buffer (10 mM Tris/Cl pH 7.5, 150 mM NaCl, 1 mM EDTA, 1% Triton X-100, 0. 5% sodium desoxycholate, protease inhibitors aprotinin 1 µg/ml, leupeptin 5 µg/ml and PMSF 0.5 mM) and centrifuged at 8000 g for 4 min. For immunoprecipitation, cells were diluted in buffer (50 mM Tris/Cl pH 7.5, 150 mM NaCl, 1 mM EDTA, 1% Triton X-100, 0.5% sodium desoxycholate, 1 mg/ml BSA, 0.5% low fat milk powder, protease inhibitors aprotinin 1 µg/ml, leupeptin 5 µg/ml and PMSF 0.5 mM) containing 5 µl anti-p18 antibody. After overnight incubation (head over tail rotation) at 4 °C, the immuno-complexes were isolated with with protein A sepharose according to standard conditions. For quantitations, the samples were subjected to SDS-PAGE and radiofluorography.

### Expression of Rab3 and homotypic fusion assay

Cells were transfected with expression constructs pcDNA3-myc-Rab3A, pcDNA3-myc-Rab3D or pcDNA3-myc-Rab3D(N135I) using a standard protocol with Lipofectamine1000 in 2×175 mm flasks. After 5 h incubation the cells were detached, pooled and plated into a 24×24 mm plate (Nunc). After 16 h incubation the cells from each plate were again removed, and pulse-labeled (20 min) in 10 ml medium containing 10 mCi [^35^S]sulphate. A PNS was prepared and resuspended in 1 ml and used for the fusion assay. Expression of the transfected proteins was measured by SDS-PAGE of equal amounts of protein, Western blotting and staining with monoclonal anti-myc antibody. The ISG–ISG homotypic fusion assay was performed as previously described [5]. In brief, complete fusion reactions are comprised of the following: 100 µl [^35^S]sulphate-labeled PNS from untransfected PC12 cells, or from PC12 cells transfected with pcDNA3-myc-Rab3A, pcDNA3-myc-Rab3D or pcDNA3-myc-Rab3D(N135I), 10 µl ISGs purified from PC12 cells stably expressing PC2, and an ATP-regenerating system were combined, incubated at 37°C for 120 min to allow fusion (30 min) and processing (90 min). The product of PC2 cleavage of SgII, which is [^35^S]sulphate-labeled p18, was immunoprecipitated and subjected to SDS-PAGE and autoradiography. The amount of p18 was quantified using ImageJ (National Institutes of Health) analysis software.

## Results

### Rab3A(N135I) and Rab3D(N135I) reduce the cortical localization of SGs

Since our previous work showed that ISGs complete their maturation in the F-actin rich cortex [2], we first screened the myc-tagged Rab3 isoforms for a potential interference with the cortical restriction of SGs. To analyze the subcellular localization of SGs, a pulse/chase-like temperature shift protocol was used to selectively label ISGs. This protocol is based on the expression of hCgB-GFP(S65T) as a marker for SGs. In brief: 24 hours after transfection, cells were incubated for two hours at 20 °C (referred to as pulse) to selectively accumulate green fluorescent hCgB-GFP(S65T) in the TGN and to block the biogenesis of ISGs. Upon release of the 20 °C block by incubation of cells at 37 °C (referred to as chase), fluorescent ISGs form at the TGN. Notably, detectable GFP-fluorescence is only generated at 20 °C and remains stable during the chase. This results in a depletion of fluorescent hCgB-GFP(S65T) in the TGN within 60–90 min. Furthermore, the length of the applied chase time correlates with the maximal age and maturation status of fluorescent ISGs [2].

**Figure 1:**
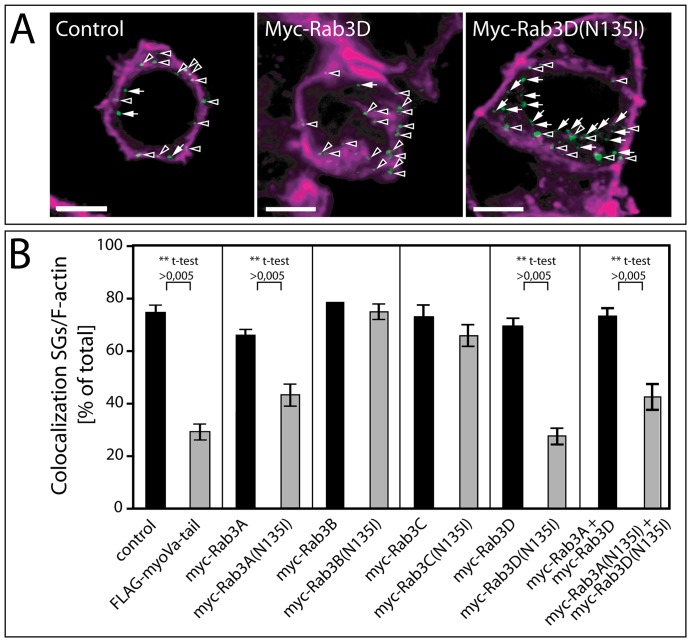
Myc-Rab3A(N135I) and myc-Rab3D(N135I) impede localization of SGs in the F-actin rich cell cortex. PC12 cells were cotransfected with hCgB-GFP(S65T) and FLAG or FLAG-MyoVa-tail, myc-Rab3A, B, C or D or their (N135I) mutants. Subsequently, cells were cultured for 2 days at 37 °C including sodium butyrate induction, and then subjected to the longer pulse/chase-like protocol with a chase time of 1 h. Cells were then fixed, stained with TRITC-phalloidin and imaged by confocal microscopy. (**A**) Representative single optical sections of cells cotransfected with hCgB-GFP(S65T) and FLAG (left), myc-Rab3D (middle) or myc-Rab3D(N135I) (right). Green, hCgB- GFP(S65T); magenta, TRITC-phalloidin; arrowheads, SGs colocalizing with F-actin; arrows, SGs not colocalizing with F-actin; scalebar, 5 µm. (**B**) Quantification of colocalization between TRITC-phalloidin and GFP. Bars, percent of colocalization; error bars, SEM (n>6 cells from at least 2 independent experiments). Unpaired two-tailed student' t-tests are indicated.

To analyze the effect of Rab isoforms and mutants on the cortical localization of ISGs, PC12 cells were cotransfected pairwise with hCgB-GFP(S65T) and the myc-tagged versions of either wild-type Rab3 isoforms or Rab3 (N135I)-mutants. Expression of all cotransfected Rab constructs was confirmed by immunofluorescence (Fig. S1). Cotransfections of hCgB-GFP(S65T) with FLAG-MyoVa-tail or FLAG were used as positive and negative controls respectively, due to their known effects on the cortical localization of SGs [3]. For each case the double-transfected cells were fixed after 60 min of chase and F-actin was stained with phalloidin-TRITC. Colocalization was analyzed by confocal 3D microscopy and subsequent image processing as described [2]. In control cells (FLAG), ∼75±2.6% (n = 9 from 3 independent experiments) of the total hCgB-GFP(S65T) labeled ISGs colocalized with cortical F-actin (Fig. 1A), whereas in cells, which coexpressed the FLAG-myoVa-tail, the number of peripheral ISGs was 29.2±3.4% (n = 7 from 3 independent experiments) (Fig. 1B), consistent with our previous findings [2], [3]. Interestingly, also the expression of myc-Rab3A(N135I) and myc-Rab3D(N135I) resulted in a strong reduction of peripheral localization of ISGs to 43. 5±3. 8% (n = 8 cells from 3 independent experiments) (Fig. 1B), and 28.0±2. 9% (n = 7 cells from 3 independent experiments) (Figs. 1A, 1B), respectively. Notably, the effect of myc-Rab3D(N135I) was as pronounced as that of FLAG-myoVa-tail (Fig. 1B). We further addressed, whether co-transfection of myc-Rab3A(N135I) and myc-Rab3D(N135I) would have a cumulative effect on the colocalization of SGs with F-actin (Fig.1B). However, the measured colocalization of 43.0±4.9% (n = 10 cells from 2 independent experiments) was similar to that of myc-Rab3A(N135I) alone and thus indicated the absence of additive effects. The expression of all non-mutated myc-Rab3 isoforms, or of the mutants myc-Rab3B(N135I) or myc-Rab3C(N135I) had no significant effect on the peripheral localization of SGs (Fig. 1B).

### Removal of bfurin is blocked by the expression of Rab3D(N135I)

**Figure 2:**
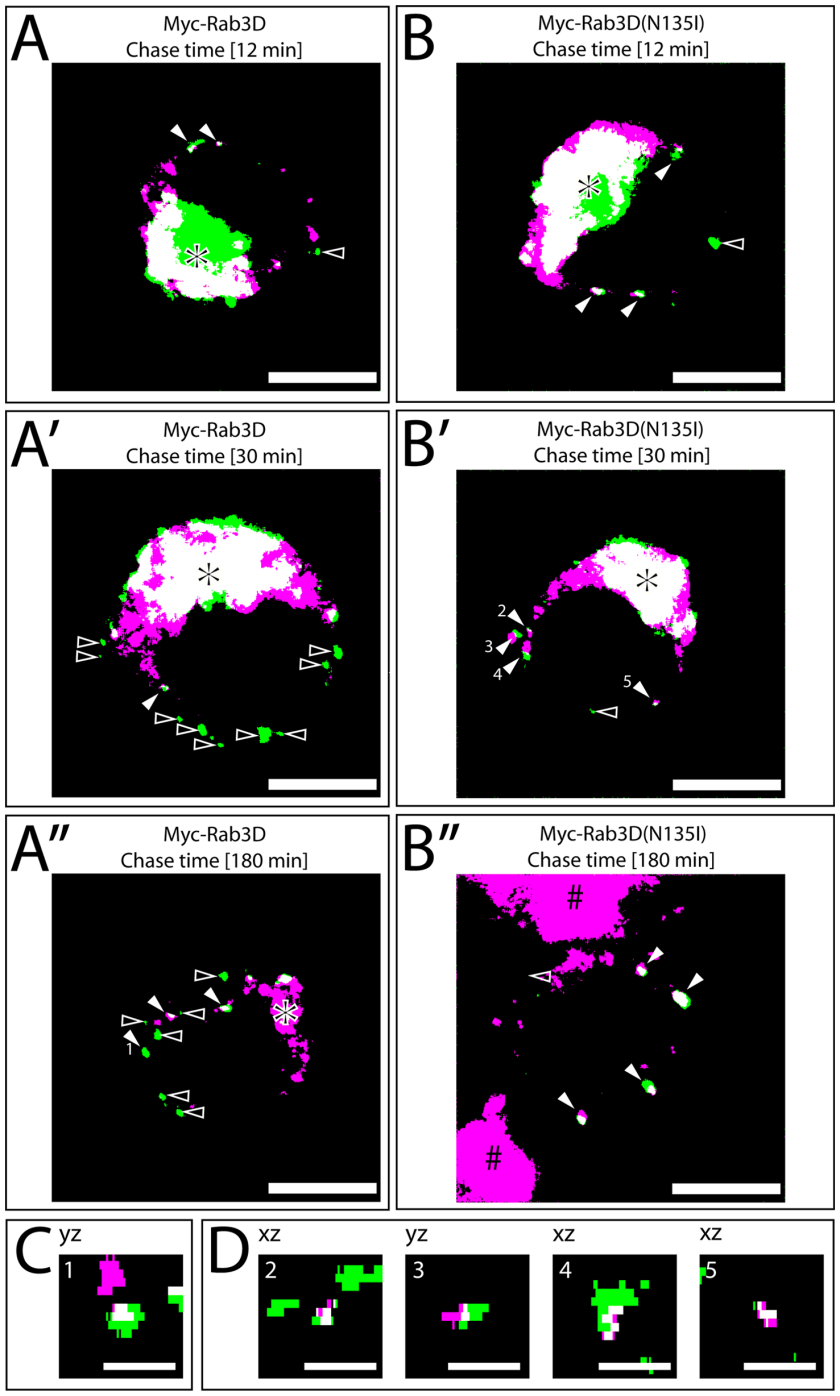
Illustration of the analysis of the colocalization of bfurin with hCgB-EGFP in 3D. Representative microscopical data used for statistical analysis (Fig. 3A). PC12 cells were triple-transfected with hCgB-EGFP, bfurin and either Rab3D (A-A″and C) or Rab3D(N135I) (B-B″, and D) and then subjected to the shorter pulse/chase-like protocol (see Experimental) applying a chase time of 12 (A,B), 30 (A′,B′,C) or 180 (A″,B″,D) min, respectively. Cells were fixed, immunostained against bfurin and imaged by 3D confocal fluorescence microscopy. Optical sections were rendered into 3D data sets, binarized and subsequently analysed for colocalization. Single optical sections display EGFP fluorescent SGs (green) and bfurin immunofluorescence (magenta) (A-B″). Filled arrowheads, SGs colocalizing with bfurin; unfilled arrowheads, SGs not colocalizing with bfurin; scalebars: 5 µm; asterisks, TGN. C,D) Side-views of five SGs from A″ or B′, respectively, correspondence as indicated by numbers 1–5 in the (x-y) planes of panel A″ and B′. Notably, in these cases colocalization is only evident in the side views. All side views of SGs shown in the Figure 2 are shown in Figure S2.

**Figure 3:**
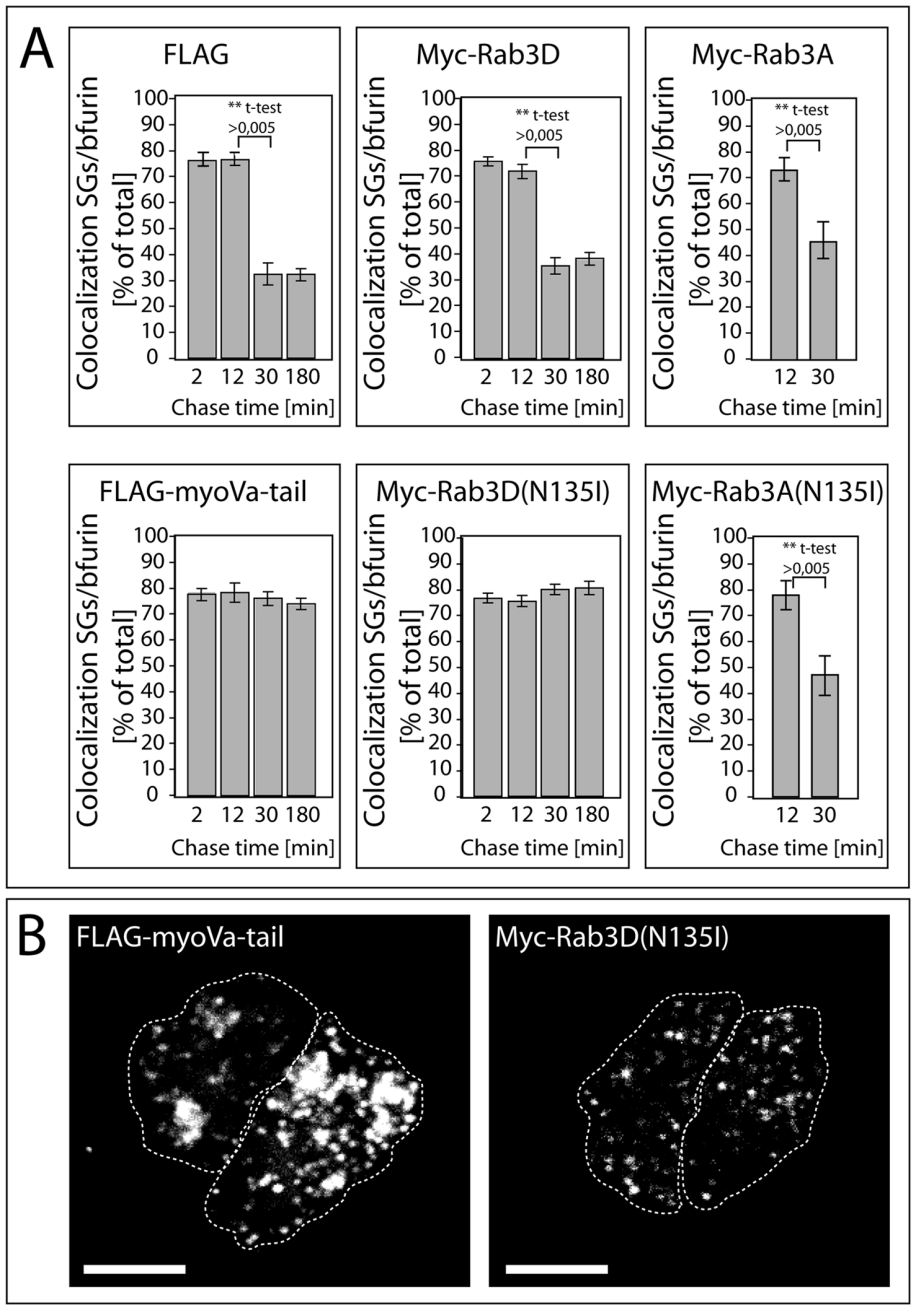
Myc-Rab3D(N135I) but not myc-Rab3A(N135I) inhibits the removal of bfurin from maturing SGs to the same extent as FLAG-MyoVa-tail. (A) PC12 cells were cotransfected with hCgB-EGFP, bfurin and FLAG, FLAG-MyoVa-tail, myc-Rab3D or myc-Rab3D(N135I) or with hCgB-EGFP, ECFP-bfurin, myc-Rab3A or myc-Rab3A(N135I). Subsequently, cells were subjected to the shorter pulse/chase-like protocol with chase times of 2, 12, 30 or 180 min, respectively, and fixed. Cells were stained against bfurin, except for cotransfections with myc-Rab3A and myc-Rab3A(N135I), imaged by confocal microscopy and analyzed for colocalization. The graphs show the percentage of hCgB-EGFP positive SGs colocalizing with bfurin signal (n = 6 cells per experiment, 2 independent experiments for myc-Rab3A and myc-Rab3A(N135I), and n≥4 cells per experiment, ≥3 independent experiments, for all other conditions); bars: mean ± SEM). Results of unpaired two-tailed student' t-tests are shown. (B) Myc-Rab3D and myc-Rab3D(N135I) do not induce clustering of SGs. PC12 cells were cotransfected with hCgB-GFP(S65T) and FLAG-MyoVa-tail, myc-Rab3D or myc-Rab3D(N135I). Cells were subjected to the long pulse/chase like protocol using a chase time of 90 min. Then, cells were fixed and imaged by confocal microscopy. The images show 3D reconstructions (Imaris) of fluorescence signals of hCgB-GFP(S65T). Scalebar: 10 µm.

To test a potential role of Rab3D and Rab3A in maturation, we analyzed whether ISGs are converted to MSGs upon expression of the respective Rab3 mutants. We first studied the removal of the endoprotease bovine furin (bfurin), which is a transmembrane protein. In PC12 cells, furin is sorted from the TGN into more than 80% of the ISGs [12]. Thereafter furin is removed from maturing SGs within 30 min [2]. Therefore, furin can be used as a marker to monitor membrane remodeling of ISGs. Because the expression level of endogenous furin was too low for immunodetection, we cotransfected PC12 cells with bfurin, hCgB-EGFP, and myc-Rab3D or myc-Rab3D(N135I). Cotransfected FLAG-MyoVa-tail was used as a positive control because of its known inibitory effect on bfurin removal [14], and cotransfected FLAG as a negative control. To perform a temporal analysis of the removal of bfurin from ISGs, transfected cells were subjected to the short pulse/chase-like protocol (see Experimental), and then fixed and immunostained against bfurin after different chase times. The colocalization of vesicles containing hCgB-EGFP and bfurin was analyzed using 3D confocal microscopy. Representative single (x-y) planes of the image stacks are shown (Fig. 2) along with the corresponding (x-z) and (y-z) views of all hCgB-EGFP positive structures (Fig. S2). This showed that 70–80% of SGs colocalized with bfurin up to 12 min of chase under all four conditions (Fig. 3A). When FLAG, myc-Rab3D, myc-Rab3A, or myc-Rab3A(N135I) were coexpressed, the colocalization decreased after 30 min of chase indicating the removal of bfurin (Figs. 2A′, S2, 3A). In contrast, when either FLAG-MyoVa-tail or myc-Rab3D(N135I) were coexpressed with hCgB-EGFP, no reduction of colocalization was observed. Instead, in both cases 70–80% of the SGs colocalized with bfurin over the entire observation period of 3 hours (Figs. 2B′B″, S2, 3A). Thus, the inhibitory effect of Rab3D(N135I) on the removal of bfurin was as potent as that of FLAG-myoVa-tail. This suggests that Rab3D but not Rab3A has a role in the membrane remodeling of maturing SGs. Since we had shown previously that FLAG-MyoVa-tail induced exhaustive clustering of SGs in PC12 cells, in addition to its inhibitory effect on the removal of bfurin [3], we investigated whether myc-Rab3D(N135I) also affected the distribution of SGs. However, no clustering of SGs above control level (FLAG, not shown) was detectable in confocal images (Fig. 3B) of myc-Rab3D(N135I) expressing cells.

### Rab3D and Rab3D(N135I) are recruited to ISGs

**Figure 4:**
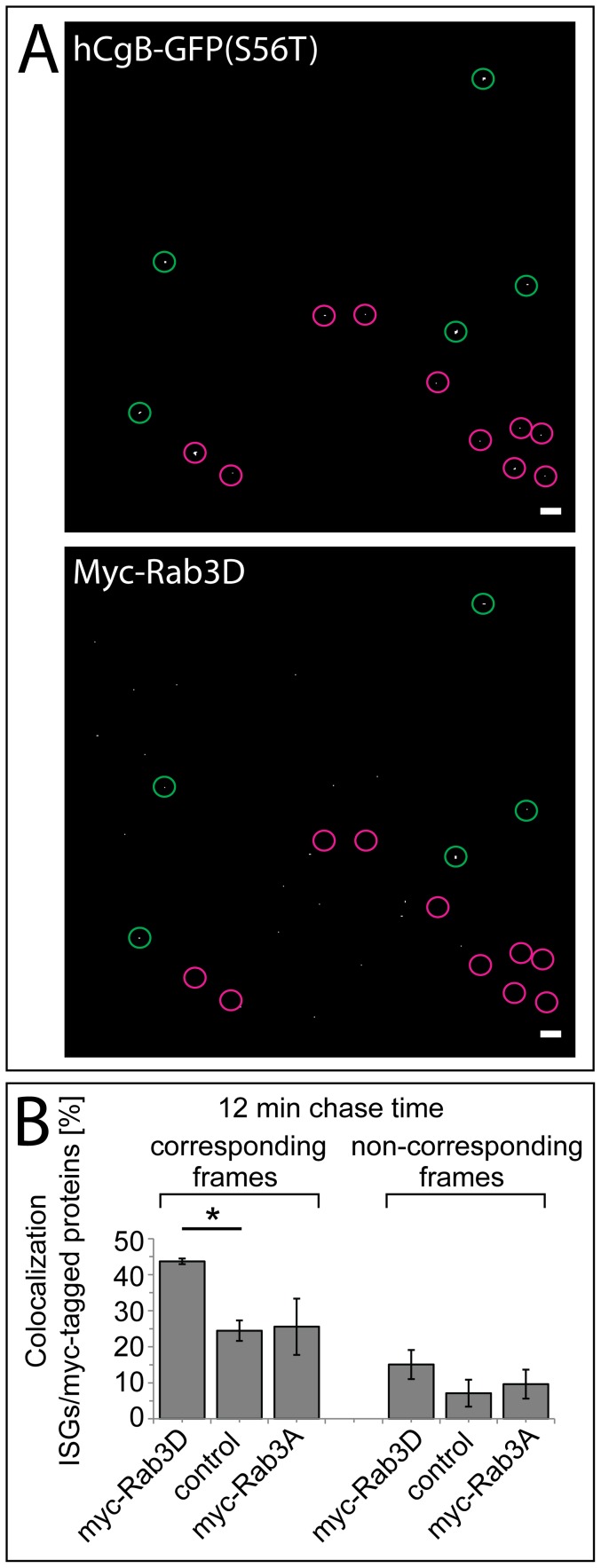
myc-Rab3D is recruited to ISGs. PC12 cells were cotransfected with hCgB-GFP(S65T) and myc-Rab3D, myc-Rab3A or control vector. Cells were cultured for 2 days including sodium butyrate induction and then subjected to the long pulse/chase-like protocol. After 12 min of chase, SGs were isolated, spun down on coverslips, fixed and stained against the myc-tag (see Experimental). (**A**) Maximum projections of processed confocal image stacks, which were used to count the percent of colocalization of spots of hCgB-GFP(S65T) signals (top) with spots of myc signals (bottom). Red circles, non-colocalizing spots, green circles, colocalizing spots; scalebars, 10 µm. (**B**) Amount of fluorescent ISGs colocalizing with myc signal in corresponding frames (left) and non-corresponding frames (right) as a control. Bars, mean ± SEM; students two-tailed t-test confidence interval: *<0,05; for each condition, ≤143 hCgB-GFP(S65T) puncta on ≤7 frames for each condition and each of 3 independent experiments. For non-corresponding frames, the green channel of all frames was paired with the red channel of the following frame.

To investigate if Rab3D is associated with maturing ISGs, we analyzed the colocalization of exogenously expressed myc-Rab3D with isolated 12 min old fluorescent ISGs. PC12 cells were cotransfected with hCgB-GFP(S65T) and myc-Rab3D, myc-Rab3A or empty vector, and subjected to the long pulse/chase-like protocol (2 h block at 20°C). After 12 min of chase, SGs were enriched by subcellular fractionation, and spun onto coverslips followed by immunostaining against the myc-epitope. Thus, only ISGs with a lifetime of less than 12 min were fluorescently labeled with GFP. Subsequently the SG layer was imaged by confocal microscopy and colocalization of GFP-fluorescence with myc-staining was analyzed. Representative microscopic images are shown in Figure 4. A statistical analysis revealed that 43.7%±0.8 of ISGs colocalized with myc-Rab3D. In contrast, Rab3A displayed a lower colocalization of 24.5%±2.9, which was comparable to the value obtained with the empty vector (25.7%±7.8) and thus reflects the background level of non-specific myc-staining (Fig. 4B). Analysis of non-corresponding frames of the two channels as a further control revealed a colocalization of 15.1%±4.1, 7.1±3.7%, and 9.6%±4.0 for myc-Rab3D, myc-Rab3A and control, respectively. In a separate set of experiments we also deteced myc-Rab3D(N135I) on newly formed SGs (Fig. S3). Thus, our data indicate a recruitment of exogenously expressed myc-Rab3D, but not myc-Rab3A, to SGs shortly after their formation at the TGN.

### Expression of Rab3D affects the buoyant density of SGs

**Figure 5:**
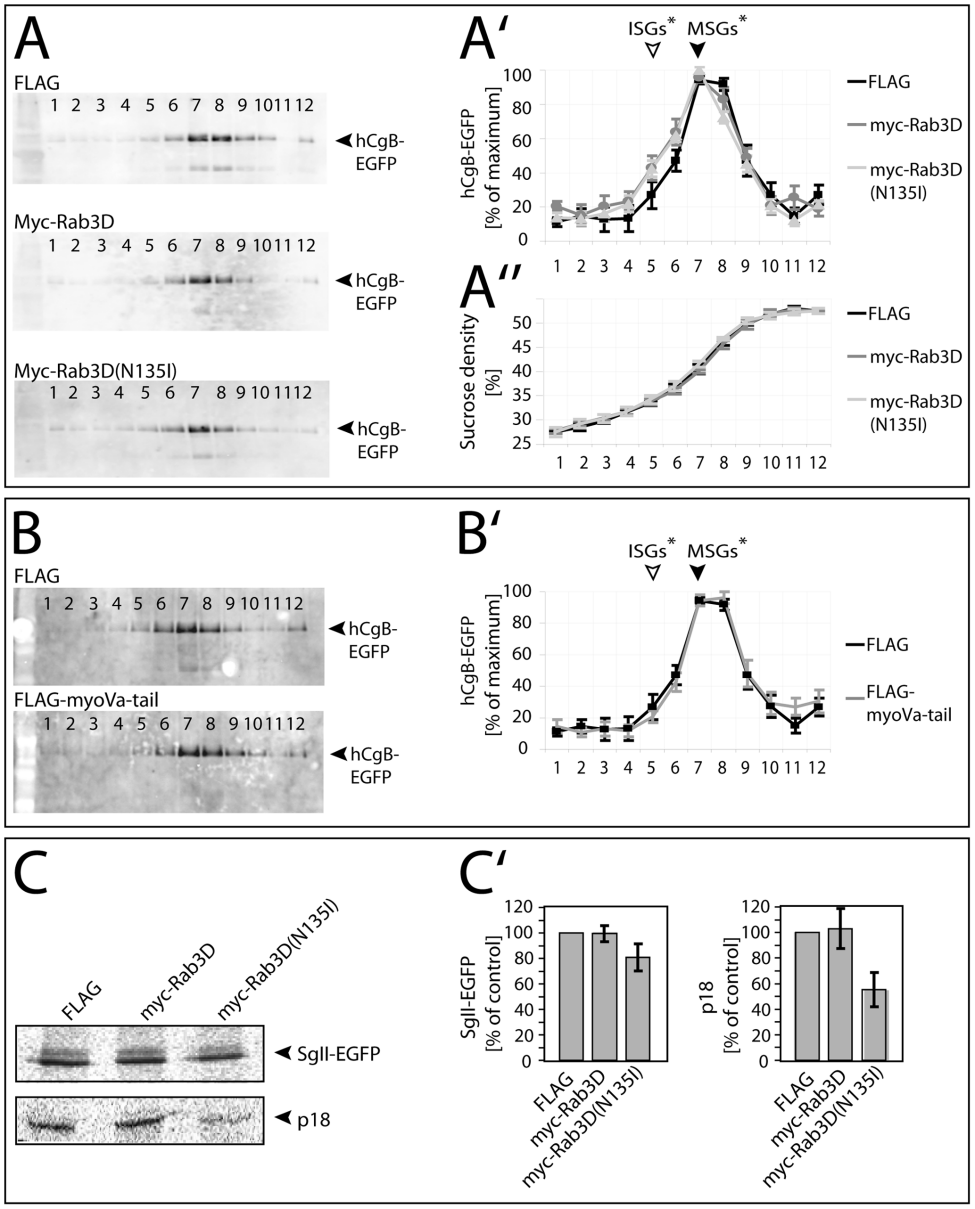
Effects of myc-Rab3D and myc-Rab3D(N135I) on buoyant density of SGs and processing of SgII. PC12 cells were cotransfected with hCgB-EGFP and FLAG, myc-Rab3D, myc-Rab3D(N135I), FLAG or FLAG-MyoVa-tail. (**A and B**) Cells were cultured for two days including sodium butyrate induction. Cell fractions enriched in SGs were analyzed by sucrose gradient centrifugation followed by Western blotting. (**A**) Western blots of one representative experiment. (**A′**) Quantification of the hCgB-EGFP signal as percent of the maximum value upon co-expression of FLAG (black squares on black line), myc-Rab3D (grey circles on grey line) or myc-Rab3D(N135I) (light grey triangles on light grey line). (**A″**) Sucrose concentrations of the respective fractions in (**A′**) are shown. (**A′**, **A″**) The published density of ISGs and MSGs [7] is indicated by unfilled and filled arrowheads, respectively. Graphs, mean ± SEM (n = 4 independent experiments) (**B**): FLAG-MyoVa-tail does not impede the maturation-dependent increase in buoyant density of SGs compared to FLAG expression only. (**B**) Representative Western blots of hCgB-EGFP upon co-expression of FLAG or FLAG-MyoVa-tail, repectively. (**B′**) Quantification of the hCgB-EGFP signals as for (**A′**) with FLAG (black squares on black line) or FLAG-MyoVa-tail (light grey line). (**B, B′**) Graphs, mean ± SEM (N = 4 independent experiments). (**C**) Expression of myc-Rab3D(N135I) impairs the processing of SgII during SG maturation. PC12 cells were cotransfected with PC2 and FLAG, myc-Rab3D or myc-Rab3D(N135I). Cells were cultured for one day including sodium butyrate induction. Then, cells were pulse-labeled with [^35^S]-sulphate for 1 hour followed by a chase of 3 hours (see Experimental). Thereafter cells were lysed, the processing product p18 (**C**, lower panel, **C′**, right panel) was immunoprecipitated and analyzed by SDS-PAGE and radiofluorography. Aliquots of the cell lysates were analyzed for endogenous rSgII (loading control C, upper panel, C′, left panel). One respresentative radiofluorography (**C, top**) for each condition and the quantitation (C′) (mean ± SEM, n = 3 independent experiments for p18, mean ± stdev, n = 2 independent experiments for rSgII) is shown.

Earlier studies demonstrated an increase in the buoyant density of SGs during their maturation [4]. To analyze whether the expression of myc-Rab3D(N135I) interferes with this increase in density, we performed sucrose density equilibrium centrifugation of SGs isolated from PC12 cells that were cotransfected with hCgB-EGFP and FLAG, FLAG-MyoVa-tail, myc-Rab3D or myc-Rab3D(N135I). Two days later SGs were enriched from PNS by subcellular fractionation and finally subjected to equilibrium centrifugation. The distribution of the SG-marker hCgB-EGFP across the gradient was determined by SDS-PAGE followed by Western blotting with an antibody specific for the GFP moiety. The exclusive detection of transfected hCgB-EGFP but not endogenous CgB ensured that only SGs from transfected cells were analyzed. Notably, the hCgB-EGFP-expressing cells were always found to be cotransfected with myc-Rab3D or myc-Rab3D(N135I) (Fig. S4). As a result, the average buoyant density of SGs was slightly lower when either myc-Rab3D or myc-Rab3D(N135I) were coexpressed, as compared to the FLAG control (Fig. 5A). This decrease was indicated by a small but significant shoulder in the hCgB-EGFP profile at ∼34. 5% sucrose (fraction number 5), which corresponds to the reported buoyant density of ISGs [4]. Under the same conditions, coexpression of FLAG-MyoVa-tail did not affect the buoyant density of SGs (Fig. 5B), which was peaking at 40. 5% sucrose (fraction number 7) in accordance with the density of SGs in non-transfected PC12 cells [4].

### Processing of SgII is impaired in Rab3D(N135I) expressing cells

We next investigated the influence of Rab3D(N135I) expression on the processing of cargo proteins in the matrix of SGs. As an example, the processing of the well known luminal marker protein secretogranin II (SgII) was analyzed. SgII undergoes a pH-dependent, proteolytic cleavage by PC2 at the level of ISGs [7]. This processing results in a final fragment of 18 kD (p18), which contains the sulfation site of SgII [7] and is therefore detectable after labeling of cells with radioactive [^35^S]sulphate. Because PC2 is not endogenously expressed in PC12 cells [7], we cotransfected PC2 with myc-Rab3D, myc-Rab3D(N135I) or FLAG control, respectively. Cells were pulse-labeled with [^35^S]sulphate, chased for three hours, lysed and then subjected to immunoprecipitation of p18. As loading control aliquots of each sample were analyzed for rSgII directly by SDS-PAGE and autoradiography before immunoprecipitation. This revealed equal amounts of rSgII for the three samples (Fig. 5C). In contrast, the amount of p18 purified from the cells transfected with myc-Rab3D(N135I) was reduced almost by half (55%±13) compared to the samples transfected with myc-Rab3D or FLAG (Figs. 5C, 5C′). Thus, cargo processing is reduced, but not blocked by expression of myc-Rab3D(N135I).

### Homotypic fusion of ISGs is not impaired in Rab3D or Rab3D(N135I) expressing cells

**Figure 6:**
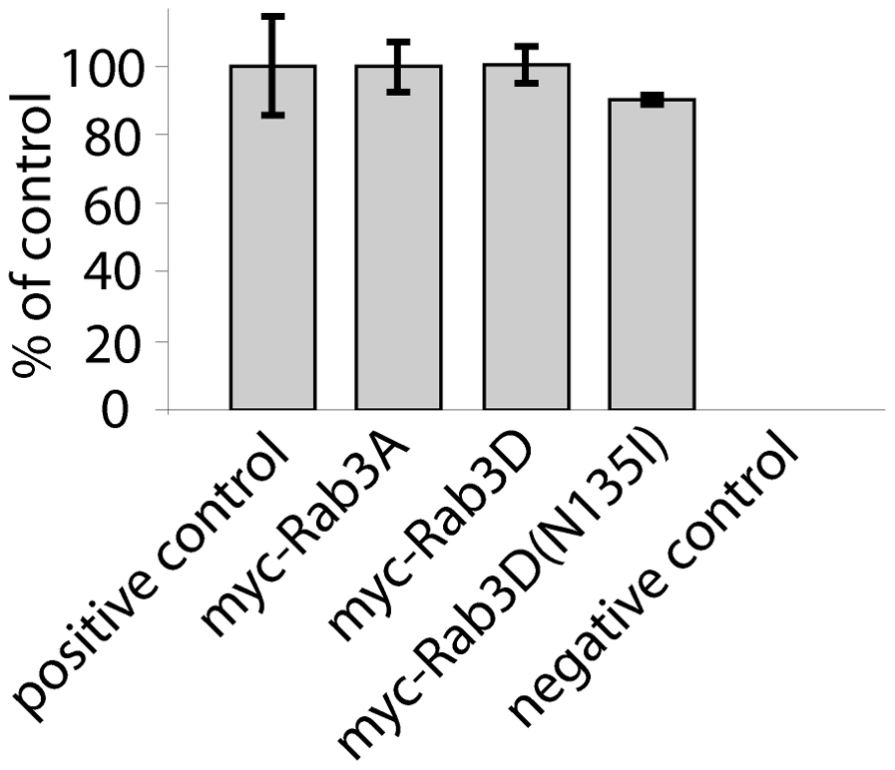
Myc-Rab3D and myc-Rab3D(N135I) do not impair homotypic fusion of ISGs. PC12 cells, untransfected or transfected with myc-Rab3A, myc-Rab3D or myc-Rab3D(N135I) were incubated for 16 h at 37°C, and then pulse-labeled for 20 min in medium containing [^35^S]sulphate. A PNS was prepared and coincubated with SGs from PC12 cells stably expressing PC2 (ISG/ISG fusion assay, [5]). The fusion was monitored by the quantitation of the amount of [^35^S]sulphate p18, a PC2-dependent processing product of SgII (see Experimental). The bar graph shows the quantification of [^35^S]sulphate-labeled p18 as a measure for homotypic fusion. p18 is expressed as percent of positive control: positive control, 100±14,4; myc-Rab3A, 99,7±7,4; myc-Rab3D, 100,2±5,2; myc-Rab3D(N135I), 90±1,4; bars: mean ± SEM, n = 3.

Since perturbed homotypic fusion of ISGs may cause impaired maturation, we investigated homotypic fusion by performing a fusion assay. PC12 cells were transfected with myc-Rab3A, myc-Rab3D or myc-Rab3D(N135I). Untransfected cells were used as a positive control (Fig. 6). After 24 hours of culturing, cells were pulse-labeled with [^35^S]sulphate for 20 minutes, and a PNS was prepared. The PNS of each condition was combined with purified ISGs from PC12/PC2 cells, which stably expressed PC2. As a negative control, PNS of untransfected cells was treated similarly, except that the PNS was not combined with purified ISGs. Fusion was analyzed by quantitation of the resulting [^35^S]-labeled p18 generated by PC2 as described previously [5] (Fig. 6B). This revealed that the degree of fusion of ISGs isolated from myc-Rab3A, myc-Rab3D or myc-Rab3D(N135I)-expressing cells was not significantly different from the value obtained with untransfected control cells (Fig. 6). Therefore, homotypic fusion of ISGs seems not to be affected by either myc-Rab3D or myc-Rab3D(N135I)-expression.

## Discussion

Our new findings show that the expression of myc-Rab3A(N135I) or myc-Rab3D(N135I) reduced the cortical restriction of ISGs (Fig. 1), whereas coexpression of both mutants did not result in an additive but smaller effect. The milder consequences observed under coexpression conditions may result from a lower expression level of each construct or may indicate some form of interaction between Rab3A and Rab3D, which counteracts the effect on cortical restriction. The same Rab3 mutants as identified here led to a reduction in cortical restriction of MSGs in PC12 cells as documented by quantitative electron microscopy [33]. Furthermore, our data show that the expression of myc-Rab3D(N135I) but not myc-Rab3A(N135I) blocked the removal of bfurin from maturing SGs (Figs. 2B, 3A). This suggests, in conjunction with results showing that furin is removed from ISGs in clathrin-coated IDVs [10], that myc-Rab3D(N135I) inhibits the formation of IDVs.

Our approach to monitor the block of furin removal by mutant Rab3D by density gradient centrifugation showed that overexpression of both myc-Rab3D or myc-Rab3D(N135I) slightly reduced the buoyant density of SGs as compared to control conditions (Figs. 5A–B). However, since only mutant Rab3D blocked furin removal but both mutant Rab3D and wt Rab3D affected the buoyant density, we speculate that the underlying reason for this reduction may be sequestration of important but limited SG maturation factors by excess Rab3D. Potential candidates for such factors are GTP/GDP-exchange factors (GEFs), which are essential for Rab3D nucleotide cycling [34]. In this respect, Kalirin and Trio, two homologous Rho GEFs, were shown to be implicated in the modulation of cargo secretion from ISGs [35]. Our conclusion that the block of furin removal caused by mutant Rab3D is not reflected by an effect on the buoyant density of SGs is further supported by our data on the role of Myosin-tail in SG maturation: although overexpression of the MyosinVa-tail mutant blocks removal of furin from SGs as strong as the Rab3D mutant, it does not lead to a detectable shift in buoyant density of SGs (Fig. 2A–C, and 4A, 4A′) [36].

In agreement with our data an involvement of Rab3D in SG maturation is further supported by several observations from studies in other cell types. In this respect, Rab3D was found to be associated with a population of SGs with low buoyant density in parotid cells [37]. Moreover, SGs of exocrine pancreas and parotid gland of Rab3D-knockout mice have an approximately doubled volume compared to SGs of wild-type littermates [38]. In addition, shrinkage of mouse zymogen granules at birth coincides with the association of Rab3D with zymogen granules [39]. Because these data suggest a role of Rab3D in determining the size of SGs, Riedel et al. proposed that Rab3D downregulates homotypic fusion of ISGs [38]. However, our *in vitro* evidence showing that neither myc-Rab3D nor myc-Rab3D(N135I) reduced homotypic fusion (Fig. 6), argues against such a role of Rab3D. Instead, the increase in SG size [38] observed in Rab3D knockout mice may result from insufficient membrane removal or reduced cargo aggregation during SG maturation.

The expression of myc-Rab3D(N135I) but not myc-Rab3D, resulted in a clear reduction of SG-specific processing of SgII (Fig. 5C). In contrast, processing of proopiomelanocortin (POMC) in AtT-20 cells expressing Rab3D(N135I) was found to be unaffected [40]. This discrepancy may result from the different experimental conditions. Whereas POMC processing was measured under steady state conditions involving endogenous proteases, our assay for SgII processing was based on a protocol involving pulse/chase-labeling with a chase time of three hours. It is therefore possible that processing in the presence of myc-Rab3D(N135I) was not blocked but only delayed due to insufficient acidification of the lumen of SGs resulting in lower activities of processing enzymes. Low enzyme activity may have been compensated with time and thus neutralized the effect of Rab3D(N135I) expression on POMC processing under steady state conditions. We speculate that insufficient acidification may be caused by retention of excess membrane in the ISG which would normally be removed in the form of IDVs. Similar to Rab3D, the GGA3 clathrin adaptor protein and synaptotagmin IV were also found to be essential for both protein removal and cargo processing [41], [42], while, similar to Myosin Va, inhibition of ARF-1-recruitment to ISGs blocked protein removal but not cargo processing [13], [43]. Therefore, intragranular maturation steps like cargo processing may depend on different mechanisms than the removal of membrane proteins.

Because the expression of FLAG-MyoVa-tail and myc-Rab3D(N135I) similarly impaired the localization of SGs (Fig. 1) and the removal of bfurin (Fig. 3), the inhibition of SG maturation of myc-Rab3D(N135I) may be achieved in concert with myosin Va. This idea of a cooperative action of myosin Va and Rab3D is consistent with our observation that both myosin Va [14] and Rab3D (Fig. 4) were already detectable on ISGs 12 min after their biogenesis at the TGN. Further support for this cooperative model is provided by the demonstration that the application of the putative myosin ATPase inhibitor butanedione monoxime (BDM) increased the number of Rab3D positive secretory organelles in alveolar epithelial type II cells suggesting a role of a myosin in the removal of Rab3D from secretory organelles [44]. Interestingly, the authors describe small Rab3D positive vesicles in proximity to secretory organelles [44], which might be the equivalent to IDVs.

In analogy to the model proposed for melanosomes [16], [17], it is conceivable that synaptotagmin-like linker proteins mediate the putative interaction of myosin Va and Rab3D. Interesting candidates for such a role include RIM2 [45] and Noc2 [46]. With respect to RIM, two isoforms have been described and evidence was obtained that both isoforms interact with Rab3 isoforms [47]. Furthermore, both RIM isoforms were shown to regulate NPY-secretion and only RIM1 but not RIM2 was shown to colocalize with Rab3A [45]. More interesting in light of our data is the study with Noc2 knockout mice, where SGs of increased size accumulated and the regulated release from insulin secreting cells was shown to be impaired [46]. This finding is reminiscent on the effects of Rab3D knockout in mice [38]. These Noc2 knockout mice displayed normal glucose levels, but under stress conditions the amount of insulin released was inappropriate and the mice became hyperglycemic [46]. Based on our data this phenomenon could be caused by suboptimal proinsulin processing. It would thus be interesting to investigate if Noc2 exerts its function in SG maturation in concert with Rab3D and myosin Va.

## Supporting Information

Figure S1 **Expression levels of the myc-Rab3 isoforms and their N135I-mutants.** PC12 cells were transfected with myc-Rab3A, B, C or D, or the respective N135I mutants. Cells were cultured for one day including sodium butyrate induction, fixed, stained against the myc tag, and imaged by wide-field microscopy. Immunofluorescence intensity was measured by the application of MatLab-based software (see Experimental). An averaged fluorescence background value of non-transfected cells was substracted. Bars, averaged myc-signal per positive cell as percentage of transfected myc-Rab3D signal per cell; error bars, SEM. The number of analyzed cells for the respective conditions ranged between 21–116 cells of at least 2 independent experiments.
(TIF)

Figure S2 **Side views of SGs shown in** **Figure 2** **.** The panels show (x-y), (x-z) and (y-z) views of hCgB-EGFP (green) and bfurin signals (magenta) of all hCgB-EGFP positive punctate structures displayed in the optical planes. Crosslines indicate the position of every individual SG in x, y and z. SG signals were classified into one of four categories as indicated: colocalizing SGs, non-colocalizing SGs below size limit, part of TGN. Colocalization of hCgB-EGFP and bfurin (white signal) is marked by an arrowhead).
(TIF)

Figure S3 **Representative images of co-transfections.** PC12 cells were double-transfected with hCgB-EGFP and myc-Rab3D or myc-Rab3D(N135I). The images (**A, B**) show that the positive cells express both markers as indicated. A statistical analysis revealed that in both cases hCgB-EGFP-positive cells always (100%) coexpressed myc-Rab3D or myc-Rab3D(N135I), respectively.
(TIF)

Figure S4 **myc-Rab3D and myc-Rab3D(N135I) are recruited to ISGs.** PC12 cells were cotransfected with hCgB-GFP(S65T) and myc-Rab3D, myc-Rab3D(N135I), or control vector. Cells were cultured for 2 days including sodium butyrate induction and then subjected to the long pulse/chase-like protocol. After 12 min of chase, SGs were isolated, spun down on coverslips, fixed and stained against the myc-tag (see Experimental). (**A**) Maximum projections of processed confocal image stacks, which were used to count the percent of colocalization of spots of hCgB-GFP(S65T) signals (top) with spots of myc signals (bottom). Red circles, non-colocalizing spots, green circles, colocalizing spots; scalebars, 10 µm. (**B**) Amount of fluorescent ISGs colocalizing with myc signal in corresponding frames (left) and non-corresponding frames (right) as a control. Bars, mean ± SEM; students two-tailed t-test confidence interval: *<0,05; **<0,005; for each condition, >322 hCgB-GFP(S65T) punctuate structures from n>28 corresponding and >27 hCgB-GFP(S65T) punctuate structures from n = 5 non-corresponding frames were analyzed from at least 2 independent experiments.
(TIF)
