# Supplementary figures and images for: Rab3D Is Critical for Secretory Granule Maturation in PC12 Cells
Source: PLoS One. 2013 Mar 19;8(3):e57321. doi: 10.1371/journal.pone.0057321 (PMC3602456; doi:10.1371/journal.pone.0057321)

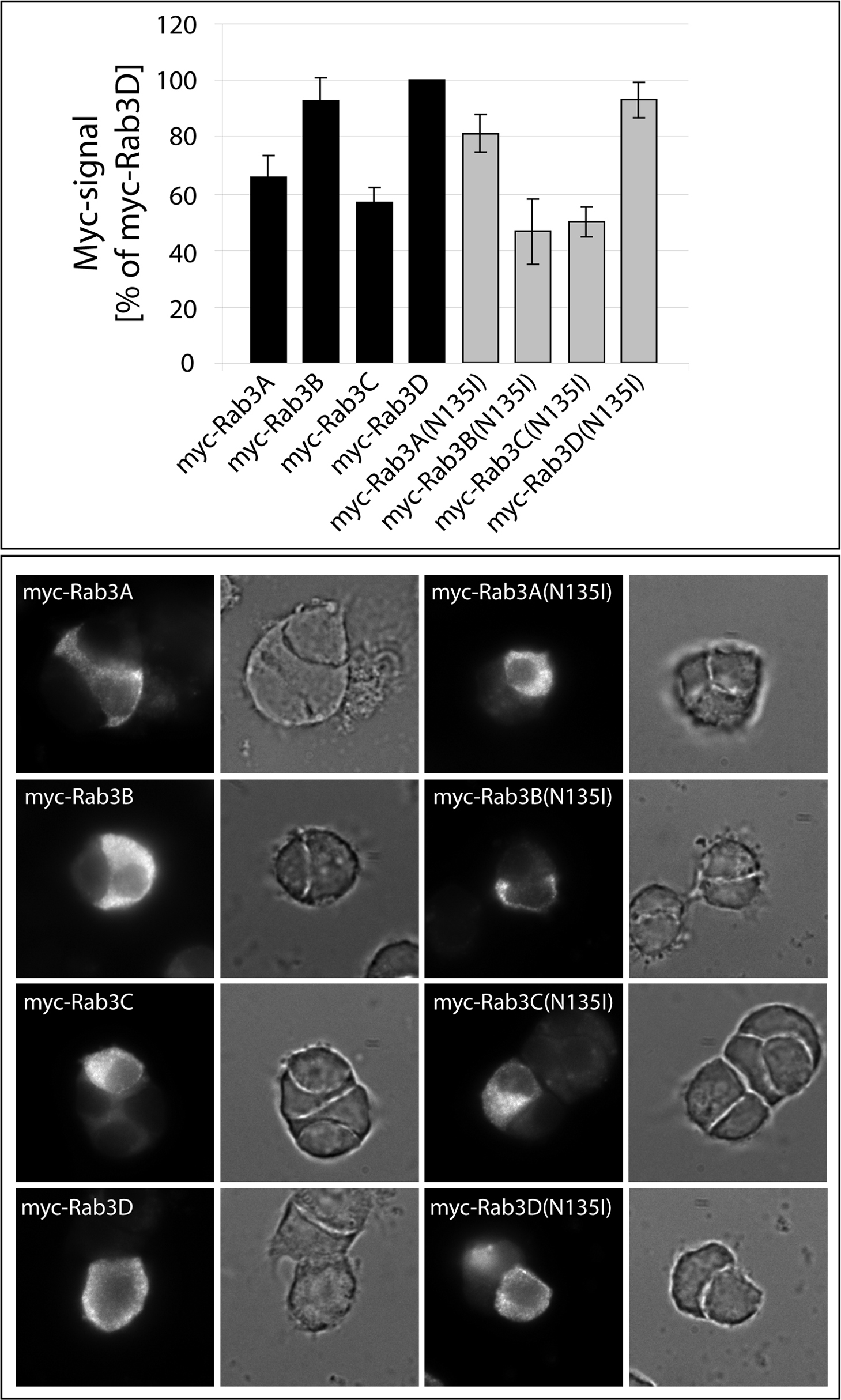

Supplement: Figure S1 — Expression levels of the myc-Rab3 isoforms and their N135I-mutants. PC12 cells were transfected with myc-Rab3A, B, C or D, or the respective N135I mutants. Cells were cultured for one day including sodium butyrate induction, fixed, stained against the myc tag, and imaged by wide-field microscopy. Immunofluorescence intensity was measured by the application of MatLab-based software (see Experimental). An averaged fluorescence background value of non-transfected cells was substracted. Bars, averaged myc-signal per positive cell as percentage of transfected myc-Rab3D signal per cell; error bars, SEM. The number of analyzed cells for the respective conditions ranged between 21–116 cells of at least 2 independent experiments. (TIF) [file pone.0057321.s001.tif]

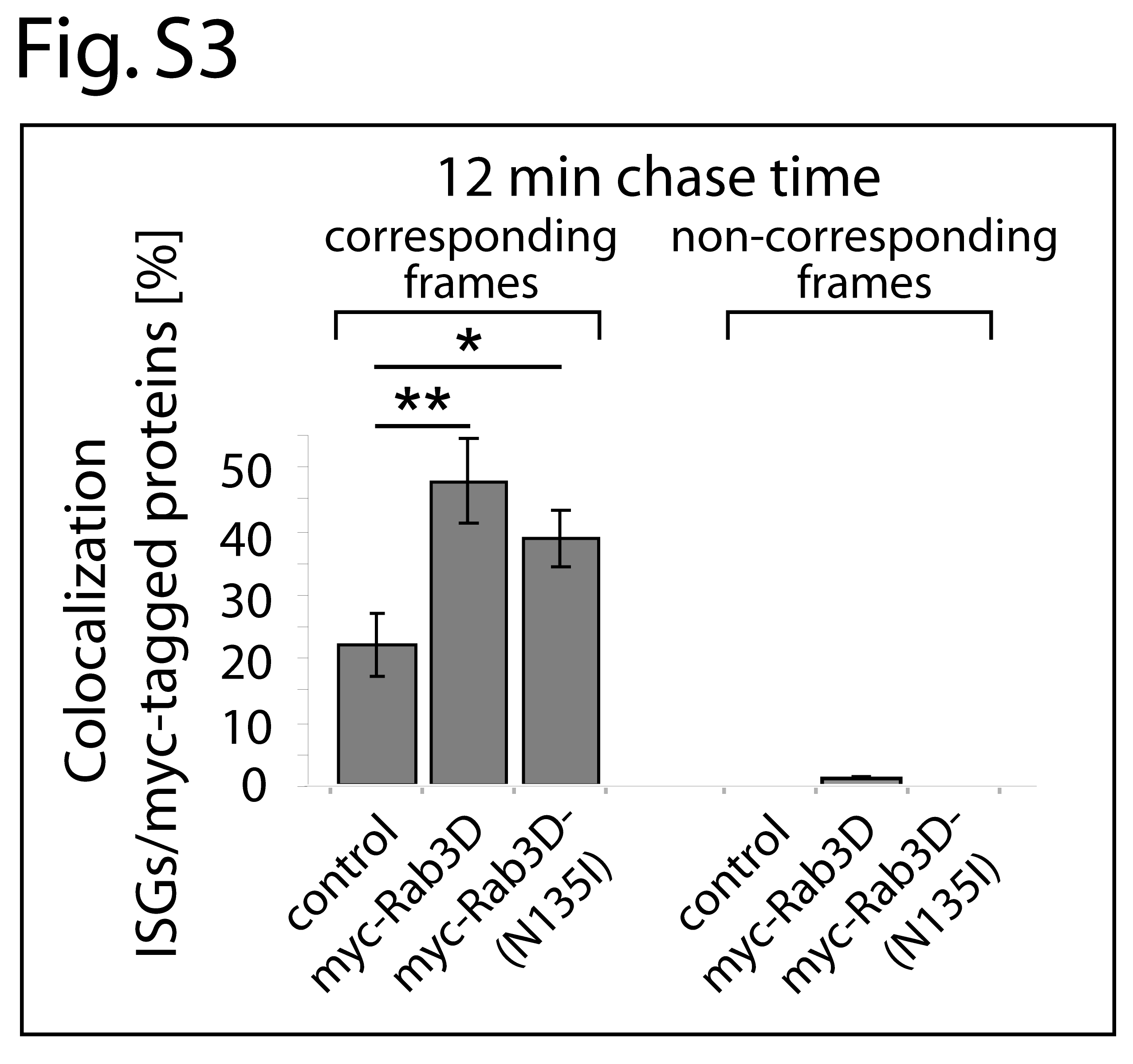

Supplement: Figure S3 — Representative images of co-transfections. PC12 cells were double-transfected with hCgB-EGFP and myc-Rab3D or myc-Rab3D(N135I). The images (A, B) show that the positive cells express both markers as indicated. A statistical analysis revealed that in both cases hCgB-EGFP-positive cells always (100%) coexpressed myc-Rab3D or myc-Rab3D(N135I), respectively. (TIF) [file pone.0057321.s003.tif]

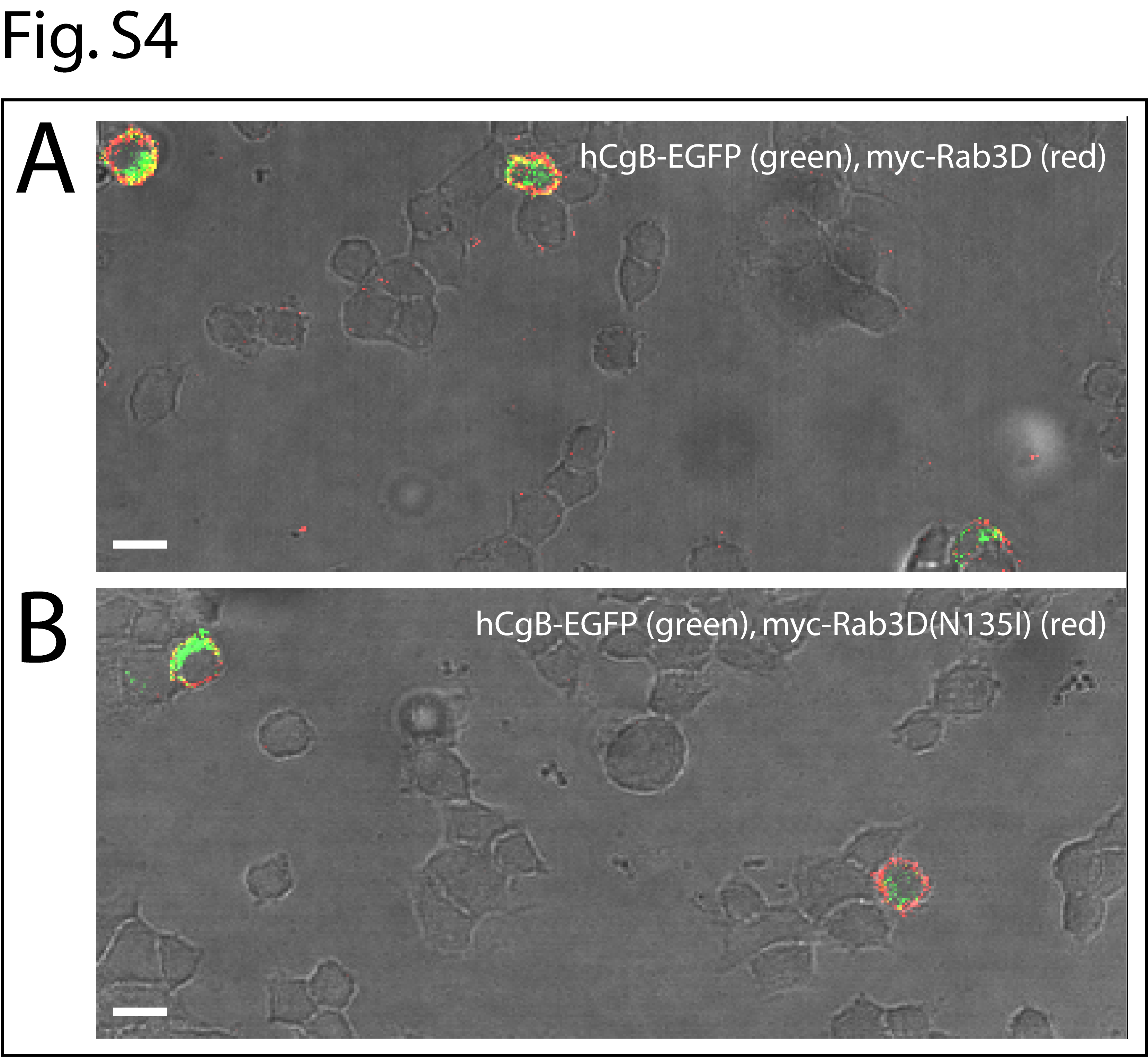

Supplement: Figure S4 — myc-Rab3D and myc-Rab3D(N135I) are recruited to ISGs. PC12 cells were cotransfected with hCgB-GFP(S65T) and myc-Rab3D, myc-Rab3D(N135I), or control vector. Cells were cultured for 2 days including sodium butyrate induction and then subjected to the long pulse/chase-like protocol. After 12 min of chase, SGs were isolated, spun down on coverslips, fixed and stained against the myc-tag (see Experimental). (A) Maximum projections of processed confocal image stacks, which were used to count the percent of colocalization of spots of hCgB-GFP(S65T) signals (top) with spots of myc signals (bottom). Red circles, non-colocalizing spots, green circles, colocalizing spots; scalebars, 10 µm. (B) Amount of fluorescent ISGs colocalizing with myc signal in corresponding frames (left) and non-corresponding frames (right) as a control. Bars, mean ± SEM; students two-tailed t-test confidence interval: *<0,05; **<0,005; for each condition, >322 hCgB-GFP(S65T) punctuate structures from n>28 corresponding and >27 hCgB-GFP(S65T) punctuate structures from n = 5 non-corresponding frames were analyzed from at least 2 independent experiments. (TIF) [file pone.0057321.s004.tif]
